# Combination of oncolytic viruses and immune checkpoint inhibitors for treatment of high-grade gliomas

**DOI:** 10.3389/fneur.2026.1683726

**Published:** 2026-02-11

**Authors:** Zhihong Qian, Qiang Gao, Wei Zhang

**Affiliations:** 1 Department of Basic Medical Sciences, School of Medicine, Tsinghua University, Beijing, China; 2 Department of Neurosurgery, Beijing Children’s Hospital, Capital Medical University, National Center for Children’s Health, Beijing, China; 3 Clinical Research Center, Beijing Children’s Hospital, Capital Medical University, National Center for Children’s Health, Beijing, China; 4 IDG/McGovern Institute for Brain Research, School of Life Sciences, Tsinghua University, Beijing, China

**Keywords:** glioblastoma, high-grade glioma, immune checkpoint inhibitor, immunotherapy, oncolytic virus, virotherapy

## Abstract

High-grade gliomas (HGG) such as glioblastoma are the most aggressive primary malignancies of the central nervous system. The median overall survival of glioblastoma is <15 months despite treatment with surgery, radiotherapy, and chemotherapy, stressing the need for additional therapeutics. Immunotherapy such as checkpoint blockade is ineffective in HGG patients owing to an immunosuppressive tumor microenvironment. Oncolytic viruses that preferentially infect and kill cancer cells represent another novel therapeutic approach and are under development for HGG treatment. We reviewed the efficacy of oncolytic viruses in HGG treatment in preclinical and clinical studies and gathered evidence suggesting the feasibility and advantage of combining oncolytic virotherapy with checkpoint blockade. We found that significant therapeutic effects of various oncolytic viruses have been validated in preclinical HGG models, but the clinical efficacy of oncolytic virotherapy alone is limited. Accumulation of tumor infiltrating lymphocytes and upregulation of immune checkpoints within tumor microenvironment following virotherapy justify the use of checkpoint inhibitors in combination with oncolytic viruses. Preliminary results indicate this combination may yield enhanced efficacy in HGG treatment. These findings suggest that oncolytic viruses combined with immunotherapy such as checkpoint blockade may have superior efficacy compared with virotherapy alone. Future studies should further assess this hypothesis using different combinations of oncolytic viruses and checkpoint inhibitors. Combined oncolytic virotherapy and immunotherapy may become an effective treatment modality to improve the survival of HGG patients.

## 1 Introduction

High-grade gliomas (HGG) are the most common primary brain malignancies in adults with rapidly progressive clinical courses (1). Glioblastoma, which is the most aggressive form of HGG, has an average annual incident rate of ~3 per 100,000 population (1). The median overall survival of glioblastoma is <15 months and the five-year survival rate is <10% (1–3), both of which are among the lowest of all cancer types. Conventional treatment for glioblastoma includes surgery, radiotherapy, and chemotherapy (i.e., with temozolomide). However, these therapies have only prolonged the median survival of glioblastoma patients by a few months (2, 4), underscoring the pressing need for novel therapeutics.

Immunotherapy has revolutionized the standard of care for various solid malignancies. Since the approvals of cytotoxic T lymphocyte associated protein 4 (CTLA-4) and programmed cell death protein 1 (PD-1) inhibitors in the early 2010s for the treatment of advanced melanoma (5), immune checkpoint blockade has been explored in many other malignancies including glioblastoma (6, 7). However, the efficacies of these drugs have not been satisfactory against glioblastoma in most clinical trials (6, 8), possibly owing to the immunosuppressive tumor microenvironment (7, 9). Attributed to poor infiltration and dysfunction of cytotoxic T lymphocytes and a relative abundance of immunosuppressive cells (e.g., regulatory T lymphocytes and M2-polarized macrophages) (10), the pro-tumor immune microenvironment in glioblastoma may blunt the immunostimulatory effect of checkpoint blockade (9). Thus, further immune modulation of the tumor microenvironment in glioblastoma might be vital to reaping the therapeutic benefit of checkpoint inhibitors.

**Figure 1:**
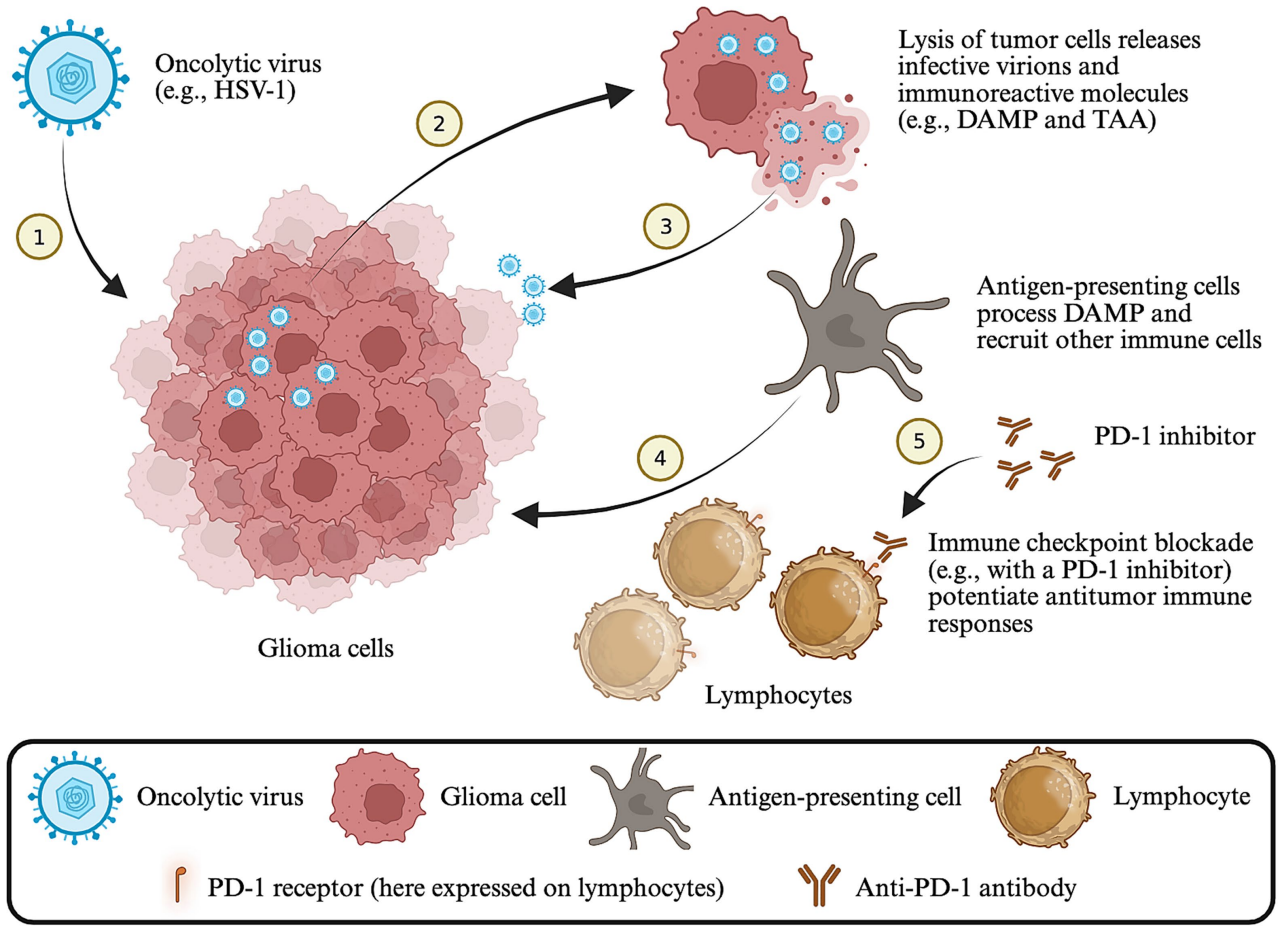
Schematic of oncolytic virotherapy for HGG. Oncolytic viruses preferentially infect and lyse glioma cells, releasing damage-associated molecular patterns (DAMP) and tumor-associated antigens (TAA). Antigen-presenting cells process DAMP and TAA, and recruit and activate tumor infiltrating lymphocytes. Immune checkpoints are upregulated within the tumor microenvironment following virotherapy (e.g., PD-1 on activated lymphocytes) and checkpoint blockade may further enhance the cytotoxic anti-tumor response. Created in BioRender. Qian, Z. (2026), https://BioRender.com/j8xvi0c.

Oncolytic viruses represent another unconventional approach to cancer treatment (11). These viruses, either naturally occurring or genetically modified, can preferentially infect and replicate in malignant cells, culminating in cellular destruction, or oncolysis (12) (Figure 1). Such oncolysis is achieved directly through completion of a lytic virus life cycle or through the action of innate and adaptive immunity of the host (12). Pathogen- and damage-associated molecular patterns and tumor-associated antigens released from lysed cancer cells can attract and activate innate immune cells, which in turn promote a tumor-specific adaptive immune response (12). Talimogene laherparepvec (T-vec), a genetically-engineered human herpes simplex virus 1 (HSV-1), was authorized for melanoma treatment in the U. S. and Europe in 2015 and has significantly improved the survival of patients with advanced melanoma (13). Meanwhile, numerous oncolytic viruses are currently under development for HGG treatment, with G47Δ (see below) authorized for clinical use in Japan in 2021 (14). Herein, we review the preclinical and clinical advances in oncolytic virotherapy for HGG treatment with a focus on viruses in clinical development that have been shown to synergize with immune checkpoint blockade *in vivo* such as HSV-1, adenovirus, reovirus, measles virus, and poliovirus (Tables 1, 2).

**Table 1:** Preclinical efficacy results of selected oncolytic viruses in HGG.

Virus type	Study model	Findings	Reference
HSV-1			
HSV1716	Orthotopic xenograft of human embryonal carcinoma NT2 cells.	Intratumoral injections of HSV1716 caused temporal tumor regression on MRI; HSV1716-treated animals survived more than twice as long as the control (25 vs. 9 weeks, *p* < 0.03).	(18)
G207	Orthotopic xenograft of U87 cells.	Intratumoral injections of G207 resulted in long-term survival (>6 months) in 15% of treated mice, whereas all mock-treated animals died within 40 days.	(22)
G47Δ	Virus replication and oncolysis was assessed *in vitro* in U87 cells. *In vivo* assays utilized athymic mice bearing subcutaneous U87 glioma.	G47Δ exhibited enhanced replicative and cytolytic activities in U87 cells than G207 *in vitro*. Intratumoral injections of 10^6^ pfu G47Δ resulted in significantly smaller tumors than G207. Complete tumor regression at 3 months in 67% of G47Δ-treated animals (vs. 25% in G207-treated, and 0% in mock-treated).	(28)
G47Δ-mIL12	Orthotopic syngeneic glioma model with 005 GSC in immunocompetent mice.	Two intratumoral injections of 5 × 10^5^ pfu G47Δ-mIL12 resulted in significantly reduced tumor cell burden (by ~50%) and extended median survival than G47Δ or mock-treated control. Survival benefit was T-cell mediated and abrogated in nude mice. G47Δ-mIL12 injections caused further reduction in tumor infiltrating CD4 + CD25 + Foxp3 + Tregs than G47Δ. G47Δ-mIL12-treated tumor cells were sorted and had decreased stem cell subpopulation and self-renewal capacity than mock-treated control in sphere formation assays. G47Δ-mIL12 injections also inhibited local VEGF expression and angiogenesis. Local IL-12 and IFN-*γ* levels in brain tumor homogenates increased on day 1 posttreatment, and IFN-γ remained elevated after 6 days.	(34)
	Orthotopic syngeneic glioma model with 005 GSC in immunocompetent mice.	Intratumoral injection of G47Δ-mIL12 led to ~2-fold increase in the number of tumor infiltrating CD3 + lymphocytes and increased iNOS+/pSTAT1 + M1-like tumor associated macrophages, with relatively stable numbers of total CD68 + macrophages, CD8 + T cells, and granzyme B + activated T cells. Triple combination of intratumoral G47Δ-mIL12 with systemic anti-PD-1 and anti-CTLA-4 resulted in long term survivors in 67–89% of the animals. Survivors were also resistant to 005GSC rechallenge on the contralateral hemisphere. Results were also validated in a more aggressive CT-2A syngeneic glioma model.	(35)
HSV-2			
OH2	Orthotopic xenograft of U87 cells or patient-derived primary glioblastoma cells in nude mice; as well as syngeneic GL261 model in C57BL/6 mice.	Two to three intratumoral injections of 3 × 10^3^–10^4^ CCID_50_ OH2 at three- to seven-day intervals significantly reduced tumor volume and extended animal survival. In immunocompetent mice, OH2 treatment caused elevated CD4 + and CD8 + T cells as well as F4/80 + macrophages within the tumors. scRNA-seq further revealed increased total tumor infiltrating macrophages and decreased M2-like tumor associated macrophages after virotherapy. Cancer cell genomic analysis revealed correlation between certain gene module expression with OH2 sensitivity.	(39)
Adenovirus			
Onyx-015	Subcutaneous patient- derived primary HGG xenograft model.	Intratumoral injections of 10^8^ pfu Onyx-015 daily for five consecutive days resulted in long-term (>4 months) tumor-free survival in 36% of *p53*-mutant tumor-bearing animals and 73% in the *p53*-wildtype group. Adenoviral replication was widespread in *p53*-wildtype tumors, but only scattered in *p53*-mutant tumors.	(42)
DNX-2401	Orthotopic xenograft of U87 cells in nude mice.	Three intratumoral injections of 7.5 × 10^5^ pfu DNX-2401 on days 3, 6, and 8 after tumor implantation led to complete tumor regression and long-term survival in 60% of the animals (vs. 15% in Delta-24 treated mice).	(46)
	Syngeneic GL261 and CT2A glioma model in C57BL/6 J mice.	DNX-2401 injection induced significant CD8 + T cell infiltration within the tumor. Levels of CD28+, ICOS+, and PD-1 + tumor infiltrating T cells increased 7–14 days after virus injection, whereas CTLA4 + and TIM-3 + expression remained low. A single intratumoral injection of 2 × 10^7^ pfu DNX-2401, followed by 3 intraperitoneal doses of anti-PD-1 antibody on days 2, 4, and 6, significantly improved median survival compared to either therapy alone and achieved 40–60% long-term survivors (vs. none in both monotherapy groups).	(47)
DNX-2440	Syngeneic GL261 and GL261-5 glioma models in C57BL/6 mice.	Intratumoral injection of DNX-2440 induced significantly more tumor infiltrating CD4 + and CD8 + T cells than DNX-2401. Three intratumoral doses of 5 × 10^7^ pfu DNX-2440 significantly extended median animal survival than DNX-2401. The survival benefits were completely abrogated in nude mice. DNX-2440 treatment increased PD-1 and PD-L1 expression within the tumor, whereas CTLA-4 expression remained unchanged. Intratumoral injection of DNX-2440 on days 6 and 10 after tumor implantation and intratumoral injection of anti-PD-L1 antibody on days 8 and 13 increased the long-term survival rate to 85%, from 28% in virus treatment alone and 15% in antibody alone. Eighty-three percent of the long-term survivors in the dual-treatment group also survived tumor rechallenge.	(55)
Ad-TD-nsIL12	Intracranial renal cancer model with Hak cells in Syrian hamsters.	A single intratumoral injection of 7 × 10^9^ vp Ad-TD-nsIL12 significantly reduced the tumor size within 6 days and resulted in 30% long-term survivors (vs 0% in mock-treated animals). Ad-TD-nsIL12 injection promoted CD3 + and CD4 + T cell infiltration within the tumor.	(58)
Reovirus			
Pelareorep	Orthotopic GL261 glioma model in immunocompetent C57BL/6 mice.	Two weeks of intravenous 5 × 10^7^ pfu reovirus with 300 ng GM-CSF per day for the first 5 days of a week, followed by three doses of intraperitoneal anti-PD-1 antibody once every 2 days on Week 3, resulted in improved survival compared to either therapy alone. Flow cytometry further showed increased IFN-γ + active CD4 + and CD8 + T cells in virus treated tumors.	(64)
Measles virus			
MV-EGFR	Orthotopic syngeneic GL261 glioma model in C57BL/6 mice.	Intratumoral injections of 2 × 10^5^ TCID_50_ measles virus on days 5, 8, 12, and 15 after tumor implantation, followed by intraperitoneal anti-PD-1 antibody on days 6, 8, and 14, significantly extended animal survival than either therapy alone and resulted in 60% long-term survivors (vs. none in other groups). The survival advantages were lost when using nude mice. Live immune cell imaging revealed increased influx of monocytes and T cells into the brain after measles virus injection. Flow cytometry further showed increased percentages of CD8 + T cells and GrB + CD8 + T cells as well as increased CD8 + T cells to regulatory T cells ratio in the dual-agent treated mouse brain than the monotherapy groups.	(68)
Poliovirus			
PVSRIPO	Syngeneic CT2A glioma model in human CD155 transgenic C57BL/6 mice for permissive virus infection. CT2A cells were also transduced with human CD155 before implantation. For survival studies, animals were pre-immunized against poliovirus before tumor cell implantation.	Intratumoral injection of 5 × 10^7^ pfu PVSRIPO temporarily reduced tumor size. Although one animal achieved complete remission, most PVSRIPO-treated tumors were comparable in size to mock-treated control by day 10 after virotherapy. Histologic examination showed marked increase and diffuse activation of microglia throughout the mouse brain and increased PD-L1 expression in Iba1 + myeloid cells after PVSRIPO treatment. Increased GzmB+ CD3 + lymphocytes, with reduced PD-1 and TIM-3 expression was observed after virus treatment. Combination of PVSRIPO and anti-PD-L1 antibody had a minor effect on median survival (24 days vs. 19 days for the control), but a significant effect on survival rate at 60 days, with a 36% remission rate. Animal with remission was also resistant to contralateral tumor rechallenge.	(73)

pfu, plaque forming units; MRI, magnetic resonance imaging; CCID_50_, median cell culture infective dose; GM-CSF, granulocyte-macrophage colony-stimulating factor; TCID_50_, median tissue culture infective dose.

**Table 2:** Clinical experiences with oncolytic viruses in HGG.

Virus type	Study design	Findings	Reference
HSV-1			
HSV1716	Phase I: Twelve HGG patients received intratumoral injection of 10^5^ pfu HSV1716.	Virus genome recovered from inoculation site in 10 patients and distal tumor sites in four. Seroconversion was detected in five patients.	(19)
	Phase I: Nine recurrent HGG patients received intratumoral injections of 10^3^–10^5^ pfu HSV1716.	No adverse clinical symptoms observed. Four patients were alive and well 14–24 months after virotherapy.	(20)
	Phase I: Twelve HGG patients received intraoperative peri-cavity injections of 10^5^ pfu HSV1716.	No clinical evidence of virotherapy associated toxicity. Three patients remained alive and clinically stable at 15, 18, and 22 months.	(21)
G207	Phase I: Twenty-one patients with malignant glioma received intratumoral injections of 10^6^–3 × 10^9^ pfu.	No clinical evidence of virotherapy associated toxicity. Reduced enhancing tumor volume in 8 of 20 patients; prolonged enhancement reduction in 2 for up to 9 months. Median progression free survival was 3.5 months. Detection of G207 genome, but not antigen, in tumor specimens on post-inoculation days 56 and 157 in 2 out of 4 patients examined. One out of five patients experienced seroconversion.	(23)
	Phase I: Nine patients with recurrent glioblastoma received stereotactic intratumoral injection of 10^9^ pfu G207, followed by focal 5 Gy radiation in 24 h; repeated virotherapy and irradiation at further progression was permitted in two patients.	Most serious adverse events were due to the underlying disease; post-inoculation seizures occurred in three patients and were possibly related to G207; transient post-inoculation pyrexia occurred in one patient. Six patients had partial response or stable disease. Median overall survival since inoculation was 7.5 months.	(24)
	Phase I: Six patients with recurrent glioblastoma received intratumoral injection of G207, followed by *en bloc* tumor resection and peri-cavity G207 injections in 2–5 days. Total inocula were 1.15 × 10^9^ pfu.	No dose-limiting toxicities were observed. Inadvertent ventricular inoculation caused transient, steroid-responsive pyrexia (39.7 °C) and altered mental status. Median progression free survival was 3 months. Median overall survival since inoculation was 6.6 months. Evidence of increased CD3+, CD8+, CD20+, and HAM56 + immune cell infiltration in tumor tissues 2–5 days after virotherapy. One patient with the longest survival had the least immune cell infiltration change, and the greatest viral replication.	(25)
	Case: A recurrent glioblastoma patient treated with intratumoral injection of 10^7^ pfu G207. Additional therapy included shorted courses of temozolomide, procarbazine, and irinotecan.	Disease- and treatment-free survival of 6 years and total survival of 7.5 years.	(26)
	Phase I: Twelve pediatric patients with supratentorial HGG received intratumoral infusions of 10^7^–10^8^ pfu G207 via 3–4 catheters, with or without 5 Gy focal radiation. Additional therapy may include temozolomide, bevacizumab, pembrolizumab, etc.	The most common adverse event associated with G207 was grade 1 pyrexia. No grade 2 or above adverse events were associated with G207. Median overall survival was 12.2 months. 5.1 months in patients with baseline HSV-1 IgG and 18.3 months in patients with seroconversion. Tumor tissue showed increased CD4+, CD8+, CD20+, and CD138 + cell infiltration months after virotherapy despite absence of virus staining.	(27)
G47Δ	Phase I/II: Thirteen patients with recurrent glioblastoma received intratumoral injections of 3 × 10^8^–10^9^ pfu G47Δ twice to identical coordinates within 2 weeks. Post-hoc immunohistochemistry revealed two cases were positive for IDH1 mutation.	The most common G47Δ-related adverse events were fever, headache, and vomiting. Median overall survival was 7.3 months; 1-year survival rate was 38.5%. Three patients (all IDH1 wildtype) survived >46 months, with one alive and stable for >11 years. Enlarging contrast enhancement on MRI following virus injection was common. Biopsy obtained during the second injection showed decreased number of tumor cells, positive HSV-1 immunostaining, and increased CD4 + and CD8 + cell infiltration. Seroconversion or increased pre-existing anti-HSV-1 IgG occurred in all patients.	(30)
	Phase II: Nineteen patients with recurrent or residual glioblastoma received up to six doses of intratumoral 10^9^ pfu G47Δ through repeated stereotactic surgery every 2–6 weeks. Virotherapy was terminated upon tumor progression, and additional treatment (e.g., re-radiation) was permitted thereafter.	Similar safety profile as in the prior trial (row above). The only grade ≥3 G47Δ-related adverse event was lymphopenia which occurred in five patients and all recovered without treatment. Median progression free survival was 4.7 months; median overall survival was 20.3 months and not affected by IDH1 mutation or MGMT status; 1-year survival rate was 84.2%. Biopsy at each repeated injection showed incrementally increased CD4 + and CD8 + lymphocytic infiltration, whereas Foxp3 + cells remained scarce.	(31)
M032	Phase I: Twenty-one patients with recurrent HGG received a single intratumoral infusion of up to 10^9^ pfu M032 via catheters.	Interim analysis: No dose-limiting toxicity was observed. Median overall survival after virotherapy was 9.38 months.	(37)
CAN-3110	Phase I: Forty-one patients with recurrent HGG received intraoperative intratumoral injection of 10^6^–10^10^ pfu CAN-3110. Dose expansion at 10^9^ pfu.	No dose-limiting toxicity was observed. Severe adverse events possibly related to virotherapy included seizures in two patients in the 1 × and 3 × 10^9^ pfu cohorts. The median overall survival was 10.9 months among recurrent glioblastoma patients and 11.6 months in the entire cohort. Positive HSV-1 serology was associated with virus clearance within the tumors and improved overall survival. Paired immunohistochemistry showed increased CD4 + and CD8 + tumor infiltrating lymphocytes after CAN-3110 treatment, which correlated with survival in HSV-1 seropositive glioblastoma patients. CD68 + CD163 + macrophages that co-expressed PD-L1 was detected after virus treatment. HSV-1 antigen was detected in a distant non-injected lesion of a multicentric glioblastoma 8 months after virotherapy.	(38)
Adenovirus			
Onyx-015	Phase I: Twenty-four patients with recurrent gliomas received intraoperative peri-cavity injections of 10^7^–10^10^ pfu Onyx-015.	No Onyx-015 related serious adverse events. The maximum tolerated dose was not reached. Median progression free time was 46 days. Median overall survival was 6.2 months. In two patients who had a re-operation 3 months after virus injections, perivascular lymphocytic and plasmacytoid cellular infiltrates were observed in the tumor.	(43)
DNX-2401	Phase I: Thirty-seven patients with recurrent HGG received one intratumoral injection of 10^7^–3 × 10^10^ vp DNX-2401. Twelve of the patients received *en bloc* resection 14 days after virotherapy for tumor tissue analysis, followed by additional peri-cavity injections of DNX-2401.	No dose-limiting toxicity. Two patients experienced DNX-2401-related adverse events including grade 1 to 2 headache, nausea, confusion, vomiting, and pyrexia. Of the 25 patients without tumor resection, the median overall survival was 9.5 months; Five patients survived for >3 years and three had a complete response for >3 years. In 12 patients who received tumor rection, the median overall survival was 13 months. Immunostaining 14 days after virotherapy revealed increased CD4 + and CD8 + T cell infiltration within the tumors. TIM-3 expression decreased in the post-treatment tissue, whereas PD-1, PD-L1, and IDO-1H expression remained stable. In one patient who had a distant, new enhancing lesion 2.5 years after DNX-2401 treatment, pathology revealed necrosis and inflammation without evidence of tumor, indicative of adaptive antitumor memory. Forty-three percent of the patients had an increased anti-Ad5 antibody titer, including 2 of the three complete responders.	(48)
	Phase I: Nineteen patients with recurrent glioblastoma received convection enhanced delivery of 10^7^–10^11^ vp DNX-2401 to tumor and surrounding brain through two catheters each. Tumor debulking was performed in five patients one week before catheter placement.	Serious adverse events possibly related to DNX-2401 infusion included grade 3 seizure in 2 patients, grade 3 increased intracranial pressure in 2 patients, and grade 4 confusion in one patient. Maximal tolerated dose was not reached. Median progression free survival was 2.7 months; median overall survival was 4.3 months. One patient had a complete response for 7.5 years. Th1-associated cytokines (IFN-γ and TNF-*α*), chemokines (e.g., IP-10, MIP-1β), CD56 + NK cells, CD4 + T cells, and CD8 + T cells all increased in CSF of most patients after virus treatment and peaked between 2 to 4 weeks. More than half patients had a considerable rise in anti-adenovirus antibody titers, which was the steepest in the complete responder.	(50)
	Phase I: Twelve pediatric patients with newly diagnosed diffuse intrinsic pontine glioma received a single intratumoral infusion of 1–5 × 10^10^ vp DNX-2401, followed by radiotherapy in ~2 weeks. Additional treatment with chemotherapy, targeted therapy (e.g., ONC201, entinostat), and immunotherapy (e.g., pembrolizumab, nivolumab) was permitted upon tumor progression or 3 months after virus infusion.	Adverse events attributable to DNX-2401 were primarily grade 1 or 2 nausea, vomiting, fever, and isolated cranial nerve palsy, except for one grade 3 neurological deterioration (e.g., bilateral oculomotor paresis and tetraparesis). Tumor size reduction on imaging occurred in 75% of the patients, independent of the virus dose. Three patients had a partial response for 3.5, 7.6, and 10.3 months. Median progression free survival was 10.7 months; median overall survival was 17.8 months. Post-treatment tumor samples were available from one patient at relapse and showed increased CD4 + and CD8 + T cell and CD163 + M2-like macrophage infiltration and reduced CD11b + myeloid cell infiltration. Single cell RNA sequencing revealed overexpression of proinflammatory cytokines (e.g., *CCL4* and *CCL3*) in tumor infiltrating macrophages after DNX-2401 treatment.	(51)
	Phase I/II: Forty-nine patients with recurrent glioblastoma received intratumoral injection of 5 × 10^8^–5 × 10^10^ vp DNX-2401. Seven days after virus treatment, pembrolizumab was administered once every 21 days for up to 2 years. Five patients had prior treatment with tumor-treating fields. Nine patients were treated with dexamethasone at baseline.	Grade 1 or 2 brain edema, headache, and fatigue were the most common adverse events related to treatment. Serious cerebral edema occurred in eight patients and was managed with dexamethasone, bevacizumab, or temporary discontinuation of pembrolizumab. No dose-limiting toxicity was observed. Objective response rate was 10.4% with two patients having a complete response and three having a partial response. Median overall survival was 12.5 months. Three patients remained alive at 45–60 months. Specific molecular features (e.g., *TP53*, *PTEN*, and *RB1* mutation) were not associated with response to treatment. Subgrouping patients based on immune cell enrichment in tumors suggested pre-treatment intermediary immune cell scores and PD-1 expression were associated with better treatment response and longer survival than patients with low or high baseline immune cell scores. Post-treatment tumor tissues from 10 patients at disease progression showed increased expression of several immune checkpoint genes (e.g., *TIGIT*, *LAG3*, and *CD276*). Changes in anti-adenovirus IgG titers were not associated with overall survival.	(53)
Ad-TD-nsIL12	Phase I: Eight patients with recurrent HGG that connected with the ventricles received 5 × 10^9^–5 × 10^10^ vp Ad-TD-nsIL12 through stereotactic injection or via an Ommaya reservoir. Four of the patients received more than one injection at a monthly interval for up to 5 doses. One patient received an PD-1 inhibitor tislelizumab twice.	Two patients in the 5 × 10^10^ vp dose group developed grade 3 generalized seizure. The maximum tolerated dose was 1 × 10^10^ vp. Two patients developed hydrocephalus that required shunt placement, but their relationship with the virus injections was unclear. The median overall survival was 5.1 months. One patient (*IDH1-*mutant and MGMT+) who received five doses of 1 × 10^10^ vp Ad-TD-nsIL12 and two doses of tislelizumab had a complete response for 18 months. Another patient with *IDH1-*mutant and MGMT+ tumor had a partial response for 3 months after two injections of Ad-TD-nsIL12. Post-treatment tumor samples were available from one patient that showed increased CD4 + and CD8 + T cell infiltration one month after a series of three virus injections. There was also a trend toward increased infiltration of M2-like CD163 + macrophages, while M1-like CD86 + macrophages remained nearly undetectable. Assessment of CSF samples from two patients revealed elevated IFN-γ, IL-10, and IL-21 after virus treatment.	(59)
	Phase I: Fifteen pediatric patients with H3K27-altered diffuse intrinsic pontine glioma received up to five intratumoral injections of 3 × 10^9^–3 × 10^10^ vp Ad-TD-nsIL12 through 1–2 Ommaya reservoir tips at diagnosis and before radiotherapy (*N* = 9) or at progression after radiotherapy (*N* = 6). Initial two virus doses were administered with a three-day interval. Follow-up injections were given every 3 weeks. Newly diagnosed patients received radiotherapy at Week 4, in place of the first follow-up virus injection. Fourteen patients received low-dose glucocorticoids and five received bevacizumab for peritumoral edema due to tumor progression or radiotherapy.	No grade 3 or above treatment-related adverse events were observed. Maximum tolerated dose was not reached. Most common virus-related adverse events were grade 1 or 2 fatigue, vomiting, and fever. Median overall survival was 10.3 months in the newly diagnosed group, and 6.4 months in patients with a progressive tumor. From disease onset, the median overall survival was 11.3 and 12.7 months, respectively, both longer than 8.3 months in a historical cohort. Post-treatment tumor samples were available from one patient upon progression and after radiotherapy that showed increased infiltration of CD68 + macrophages 2 months after virotherapy. CD163 + cells increased, but not significantly, and CD86 + cells remained low, suggesting a predominantly M2- like phenotype. CD3, CD4, and CD8 expression did not change significantly in this sample.	(60)
Reovirus			
Pelareorep	Phase I: Twelve patients with recurrent malignant gliomas received intratumoral injections of reovirus at 10^7^–10^9^ TCID_50_. Six patients underwent tumor re-resection.	No grade 3 or 4 treatment-related adverse events were observed. Maximum tolerated dose was not reached. Median time to progression was 4.3 weeks. Median overall survival was 21 weeks. Seroconversion occurred in 10 patients. Focal collections of plasma cells were identified in half of the post-treatment tumors examined.	(62)
	Phase I: Fifteen patients with recurrent HGG received intratumoral reovirus at 10^8^–10^10^ TCID_50_ through a 72-h infusion.	One grade 3 seizure was possibly related to virus treatment, but dose-limiting toxicity was not identified and maximal tolerated dose was not reached. Median time to progression was 8.7 weeks. Median overall survival was 20 weeks.	(63)
	Phase I: Nine patients with HGG or brain metastasis (i.e., colorectal cancer and melanoma) received a single intravenous infusion of 10^10^ TCID_50_ over one hour three to 17 days before surgical resection of the tumor.	Virus infusion was well tolerated in all cases. The most common adverse events were lymphopenia (grade 3–4 in six patients) and flu-like symptoms. Median overall survival was 469 days. Peripheral CD14 + monocytes, CD19 + B cells, CD56 + NK/NKT cells, and granulocytes were associated with reovirus RNA and may be responsible for virus carriage. Significant elevation in serum IFN-α and other cytokines (e.g., IL-3, M-CSF) was observed 2 days after infusion. Immunohistochemical staining for reovirus σ3 capsid protein was positive in 67% of the tumors, and immunogold staining was positive in all patients under transmission electron microscopy. RNA sequencing of post-treatment HGG tumors showed an eight-fold increase in *CCL3* and *CCL4* expression compared to non-trial control tumors. Functional analysis discovered enrichment in programmed cell death, regulation of viral transcription, and cytokine activity. Immunostaining also revealed increased tumor infiltration of CD3 + and CD8 + T cells and CD68 + macrophages, and increased expression of PD-1 and PD-L1.	(64)
	Phase I: Six pediatric patients with recurrent or progressive high-grade brain tumors received 2 days of subcutaneous sargramostim (GM-CSF), followed by 3 days of intravenous Pelareorep at 3–5 × 10^8^ TCID_50_. The treatment cycle was repeated after 28 days if no dose-limiting toxicity was observed. Three patients received concurrent dexamethasone.	One patient had a dose-limiting toxicity of hyponatremia, but maximum tolerated dose was not reached. Grade 3 or 4 adverse events included leukopenia, hypophosphatemia, confusion, and depressed consciousness. Median time to progression was 32.5 days; Median overall survival was 108 days. Seroconversion occurred in both patients tested.	(66)
Measles virus			
MV-CEA	Phase I/II: Twenty-two patients with recurrent HGG received peri-cavity injections of 1 × 10^5^–2 × 10^7^ TCID_50_ MV-CEA with (*N* = 13) or without (*N* = 9) an additional intratumoral virus injection 5 days prior to surgery. Baseline anti-measles virus immunity was required.	No dose-limiting toxicity or grade 3–4 treatment-related adverse event was observed. Maximum tolerated dose was not defined. Median progression free survival was 3.4 months. Median overall survival was 11.6 months. Immunostaining showed significantly increased infiltration of CD8 + and CD68 + cells, but not of CD4 + cells, in matched tumor samples 5 days after virotherapy. Anti-measles virus antibody titers remained constant 4 weeks after virotherapy.	(69)
Poliovirus			
PVSRIPO	Phase I: Sixty-one patients with recurrent supratentorial grade 4 glioma received a single, convection enhanced infusion of PVSRIPO at 10^7^–10^10^ TCID_50_ over 6.5 h. None of the patients received further tumor resection at the time of virus treatment. Additional treatment (e.g., lomustine and irinotecan) was permitted at disease progression.	One dose-limiting toxicity (intracranial hemorrhage) occurred at 10^10^ TCID_50_. Sixty-nine percent of the patients had a grade 1 or 2 PVSRIPO-related adverse event, and 19% had a PVSRIPO-related adverse event of grade 3 or higher. Median overall survival was 12.5 months (vs. 11.3 months in the historical cohort). Overall survival at 24 and 36 months was both 21% (vs. 14 and 4% in the historical cohort).	(74)
Newcastle disease virus			
MTH-68/H	Case series: Fourteen HGG patients, including children and adults, received intravenous MTH-68/H of up to 8 × 10^7^ pfu daily. Dosing was then individually adjusted and maintained at 4 × 10^7^–2.5 × 10^8^ pfu two to three times a week.	Seven patients remained alive at the time of report, four of whom survived for 4–7 years without any additional cancer treatment. No treatment-related adverse event was observed in any patient.	(76)
Parvovirus			
ParvOryx	Phase I/IIa: Eighteen patients with recurrent glioblastoma received a single intratumoral injection or five consecutive daily intravenous infusions of 10^6^–5 × 10^9^ pfu ParvOryx, followed by tumor resection and peri-cavity virus injection 9 days after the first virus dose. Concurrent treatment may include temozolomide, bevacizumab, and irinotecan.	No adverse event was directly attributable to ParvOryx. Maximum tolerated dose was not reached. Median progression free survival was 111 days. Median overall survival was 464 days. ParvOryx dose or administration route did not affect survival. *In situ* hybridization showed viral genome in tumor areas distant from catheter tips. After intravenous injections, viral genome was detected in 50% of the tumors, but viral protein NS1 was detected in none. CD8 + and, to a lesser extent, CD4 + T cells were the predominant immune cell infiltrates in the tumor after virus treatment. Positivity for granzyme B and perforin indicated T cell activation. Foxp3 + Tregs were scarce.	(77)

pfu, plaque forming units; vp, virus particle; CSF, cerebrospinal fluid; TCID_50_, median tissue culture infective dose.

## 2 Results

### 2.1 Herpes simplex virus 1/2

HSV-1, which has a natural affinity for neural tissue, is among the best characterized oncolytic viruses in HGG treatment. In 1991, a thymidine kinase deficient HSV-1, denoted as dlsptk, was found to be effective in killing patient-derived glioblastoma cell cultures and extending the survival of nude mice bearing U87 glioma (15). However, neurotoxicity was a concern since dlsptk did not respond to standard HSV-1 treatment such as acyclovir (16). In contrast, hrR3, a ribonucleotide reductase defective HSV-1, inhibited the growth of glioblastoma in nude mice while retaining responsiveness to acyclovir treatment (17). Another first-generation oncolytic HSV-1, HSV1716, is attenuated through deletion of genes that encode the neurovirulence factor infected cell protein (ICP) 34.5. In an NT2 glioma murine model, the survival time of tumor-bearing animals more than doubled after treatment with HSV1716 (18). In a series of two phase I clinical studies, it was demonstrated that HSV1716 was able to replicate in glioblastoma following intratumoral injection (19), and the treatment was overall well-tolerated (20). A third trial followed up with 12 HGG patients who received HSV1716 injection into surgical cavities immediately after maximal tumor resection (21). Although most patients still perished within a year after virotherapy, two survived 15 and 22 months without any signs of tumor progression by the time of report, and another was clinically stable at 18 months with additional chemotherapy and surgery (21).

G207 is a double-attenuated oncolytic HSV-1 that is both ICP34.5 and ribonucleotide reductase deficient (22). In a U87 glioma murine model, 15% of the animals that received intratumoral administration of G207 survived long-term, while the control group all died (22). Intralesional injection of G207 either as a stand-alone therapy or in combination with irradiation was found to be tolerable in patients with HGG, and radiographic evidence suggestive of oncolytic activity was observed in some cases (23, 24). While patients’ median survival since virus inoculation was ~7 months in these clinical trials (23–25), Whisenhunt et al. reported 6 years of disease-free survival in a recurrent glioblastoma patient who received G207 treatment in addition to conventional therapy (26). In 2021, G207 treatment with or without radiotherapy was tested in 12 pediatric HGG patients (27). The median survival following virotherapy was 12.2 months, in contrast to a historical baseline of 5.6 months (27). Importantly, comparisons between tissue samples pre- and post-virotherapy revealed a substantial increase in the number of CD3+, CD4+, and CD8 + tumor infiltrating lymphocytes (27), indicating a treatment-induced antitumor immune response.

G47Δ was designed based on G207 with an additional deletion in gene *US12* which encodes ICP47 (28). Lack of ICP47 in G47Δ enhances the presentation of viral epitopes on infected cells via major histocompatibility complex class I and stimulates CD8 + T cell mediated cytotoxicity (28). Coincidentally, deletion of US12 shifts the promotor for gene *US11*, resulting in more productive viral replication than G207 in human glioblastoma cell lines (28). G47Δ was shown to be more effective than G207 in inhibiting tumor growth and prolonging animal survival in a U87 murine model while retaining a similar safety profile (28). The efficacy and safety of G47Δ treatment in recurrent glioblastoma patients were validated in two phase I/II clinical trials (UMIN000002661 and UMIN000015995) (29), with post-treatment oncolytic responses and intratumoral CD4 + and CD8 + T cell infiltration confirmed on histology (30, 31). G47Δ was subsequently approval for clinical use in Japan in June 2021 (14). A retrospective regulatory analysis of the trial data raised the concern that the reported median overall survival of 20.2 months might have been caused by a higher proportion of IDH-mutant tumors (32%), but ultimately supported potential efficacy of G47Δ based on the extended periods of stable disease in some patients, supported by radiographic evaluation (32).

G47Δ was further modified through insertion of the gene encoding murine interleukin 12 (mIL12), generating G47Δ-mIL12 (33, 34). Compared with G47Δ treatment, G47Δ-mIL12 decreased tumor burden and further extended the survival of immunocompetent mice bearing syngeneic tumors derived from 005 glioblastoma stem cells (GSC) (34). The 005 GSC derived murine glioma is poorly immunogenic and recapitulates glioblastoma in human patients (34). Interestingly, M1 polarization of tumor associated macrophages and an increased ratio of effector T cells to regulatory T cells were observed in tumors after treatment with G47Δ-mIL12, suggesting an immunostimulatory effect (35). Whereas G47Δ-mIL12 alone did not cure any animals, the use of dual CTLA-4 and PD-1 inhibitors synergized with G47Δ-mIL12 therapy, which resulted in complete remission and long-term survival in up to 89% of the animals tested (35). Survivors were also resistant to 005 GCS rechallenge, indicating a durable anti-tumor immune response (35). While G47Δ-mIL12 has not been translated into the clinic, an HSV1716-based oncolytic HSV-1 expressing human IL12, named M032, with a potentially greater immunostimulatory effect and therapeutic efficacy than its parental virus has been evaluated in a phase I clinical trial for HGG treatment (NCT02062827) (36). Detailed descriptions on study findings have not been published, but an interim report stated an acceptable safety profile with a median survival of 9.38 months (37). A trial to assess safety of combined M032 and pembrolizumab in recurrent HGG patients is currently recruiting (NCT05084430).

CAN-3110 is an HSV-1-based oncolytic virus that retains ICP34.5 expression (38). Particularly, the neurovirulence factor ICP34.5 is under the transcriptional control of a nestin promoter, which is overexpressed in many malignancies including glioblastoma, but not in normal brain tissue. In a first-in-human phase I trial among 41 patients with recurrent HGG, grade 3 seizures requiring hospitalization and intervention occurred in two patients, but dose-limiting toxicities or virus-induced meningitis/encephalitis were not observed, which indicates an overall amenable safety profile (38). The median overall survival was 11.6 months in the entire cohort and 10.9 months in patients with recurrent glioblastoma. Interestingly, the trial also found that seropositivity against HSV-1, both at baseline and following virotherapy (i.e., seroconversion), pre-treatment seropositivity-dependent polyclonal T cell expansion, and expedited viral clearance within tumors were associated with improved overall survival (38), suggesting an immunological mechanism underlying therapeutic responses. Paired pre- and post-treatment sampling of tumor specimens revealed diffusely increased CD4 + and CD8 + tumor infiltrating lymphocytes with relative clustering in the perivascular space and around necrotic tumor tissues (38), suggesting immune activation following oncolysis. CD68 + CD163 + macrophages expressing PD-L1 were also detected in post-treatment tumors.

OH2, derived from herpes simplex virus 2, has recently been shown cytotoxic to glioblastoma cells and intratumoral delivery suppressed tumor growth and prolonged survival in nude mice bearing orthotopically-implanted patient-derived glioblastoma stem cells (39). In immunocompetent mice inoculated with GL261 cells, single-cell RNA sequencing revealed increased effector CD4 + and CD8 + T cell infiltration, accompanied with a decrease in tumor associated M2 macrophages and naïve CD4 + T cells following virotherapy, suggesting activation of innate and adaptive immune response (39). A phase I/II trial assessing the tolerability of intratumoral OH2 injections in patients with recurrent central nervous system tumors and preliminary effectiveness in recurrent glioblastomas is currently recruiting (NCT05235074).

### 2.2 Adenovirus

Human adenovirus type 5, from which many oncolytic adenoviruses derive, typically causes lytic infection in respiratory and gastrointestinal tracts (40). To support oncotropism, a segment was deleted in the *E1B* gene of a type 2/5 chimeric adenovirus, giving rise to Onyx-015. This modification restricted Onyx-015-mediated oncolysis to *p53*-defective glioblastoma cells *in vitro* (41), but it was later found that Onyx-015 could also induce significant tumor regression and long-term survival in mice bearing subcutaneous *p53*-intact HGG xenografts from patients (42). In a phase I clinical trial, injection of Onyx-015 into surgical cavities was shown to be safe in recurrent HGG patients, but the median survival of those diagnosed with glioblastoma was still merely 4.9 months (43).

Delta-24 is a type 5 adenovirus with a 24-bp deletion in *E1A* gene such that it preferentially infects cells with a dysfunctional p16/RB/E2F pathway (44), a frequent feature in glioma (45). Delta-24-RGD, also known as DNX-2401, was constructed based on Delta-24 through insertion of an Arg-Gly-Asp peptide motif into adenovirus fiber to enhance cancer cell binding (46). Compared with Delta-24, DNX-2401 treatment increased the rate of long-term survival from 15 to 60% in athymic mice bearing U87 glioma (46). In immunocompetent mice bearing syngeneic GL261 glioma, elevated expression of PD-1 was observed on tumor infiltrating lymphocytes following DNX-2401 treatment, and addition of a PD-1 inhibitor potentiated the therapeutic effect of DNX-2401 (47). In a phase I clinical trial, intratumoral injection of DNX-2401 in recurrent HGG patients was safely tolerated (48). Tumor reduction was observed in 18 out of 25 patients based on radiographic findings, and the median survival was 9.5 months after virotherapy (48). Notably, three patients experienced >95% reduction in tumor size, and five survived >3 years after virotherapy (48). Infiltration of CD8 + and T-bet+ cells was found in tumors resected 14 days after DNX-2401 treatment in a parallel group of patients, which suggests an activated regional immune response (48). In two separate studies, pro-inflammatory cytokines were detected in cerebrospinal fluid of DNX-2401-treated glioblastoma patients, indicative of a systemic immune reaction (49, 50). In pediatric patients with primarily H3K27-altered diffuse midline glioma, intratumoral infusions were also tolerated, with histologic evidence of increased infiltration of CD4 + and CD8 + T cells and CD163 + M2 macrophages (51). Combination of DNX-2401 and a PD-1 inhibitor (i.e., pembrolizumab) has been evaluated in 49 patients with recurrent glioblastoma (NCT02798406) (52). The combination was found well-tolerated. Pembrolizumab was discontinued in four patients due to brain edema, but was resumed following edema resolution. The objective response rate was 10.4%, with a median duration of 9.4 months, and 2 patients had a complete response for more than 2 years (53). Additionally, stable disease was observed in 45.8% of the patients. The median overall survival was 12.5 months, greater than that in earlier trials with either DNX-2401 (9.5 months) or PD-1 blockade (9.8 months) alone (48, 54). Characterization of pre-treatment tumor specimens revealed that expression of *PDCD-1* that encodes PD-1 was associated with reduction in tumor size and extended survival.

Delta-24-RGDOX, also known as DNX-2440, was designed based on DNX-2401, but expresses OX40L, the immune co-stimulatory ligand for OX40 receptors on T cells (55). Compared with DNX-2401, DNX-2440 treatment induced greater extent of lymphocytic infiltration and led to a long-term survival rate of 28% in immunocompetent mice bearing GL261-5 glioma (55). The long-term survival rate further rose to 85% when the virotherapy was supplemented with immune checkpoint blockade (55). Five of the six long-term survivors also tolerated tumor rechallenge in the contralateral cerebral hemisphere (55), suggesting the presence of immunological memory. A clinical trial of DNX-2440 treatment in recurrent glioblastoma patients was initiated in 2018 (NCT03714334), but no results have been disclosed.

Ad-TD-nsIL12 is an oncolytic adenovirus with triple gene deletions in *E1ACR2*, *E1B19K*, and *E3gp19K* and insertion of a non-secretory interleukin 12-encoding gene (56). Importantly, compared to traditional human adenovirus type 5-based constructs, Ad-TD-nsIL12 retains *E3B* expression, which may help induce sustainable viral replication in tumor cells and reduce immunosuppressive macrophage infiltration (57). In a hamster brain tumor model of orthotopically implanted renal cancer cells, intratumoral injection of Ad-TD-nsIL12 significantly reduced the tumor size within 6 days, and achieved long-term survival in 30% of the animals compared to 0% in the control group (58). Importantly, viral replication within the tumors induced alterations of the immune microenvironment, with increased CD3 + and CD4 + T cell infiltration. Sampling of the normal brain, liver, lungs, kidneys, heart and spleen tissues also excluded off-target viral replication, suggesting an excellent safety profile. In a phase I trial among eight recurrent high grade glioma patients with ventricle invasion, virus administration of up to 10^10^ particles through an Ommaya access or intratumorally was found to be safe, with only low-grade adverse events including fever, cognitive disturbances, and isolated minor seizures (59). Complete or partial response for up to 18 months was observed in two patients with IDH-mutant HGG. In 15 pediatric patients with H3K27-altered diffuse midline glioma, early pre-radiation intratumoral virus injection resulted in a 33% objective response rate and 89% disease control rate (60). Virotherapy extended the median overall survival to 11.3–12.7 months since diagnosis, compared to 8.3 months in the historical cohort, although multifocal viral injections were required in 40% of the patients.

### 2.3 Reovirus

Pelareorep, previously designated as Reolysin, is an unmodified type 3 reovirus that preferentially replicates and promotes cytolysis in Ras-pathway-activated cancer cells such as glioblastoma cells (61). In the first two clinical trials, intratumoral administration of Pelareorep was proven safe in patients with recurrent HGG, but the median survival following virotherapy was still <5 months (62, 63). Samson et al. demonstrated that intravenous inoculation of Pelareorep, a largely simplified procedure compared with intracranial administration, could also deliver the virus to tumors in HGG patients (64). Systemic increases in immunostimulatory cytokines, augmented infiltration of cytotoxic T cells and macrophages, and upregulated expression of PD-1 and its ligand, PD-L1, within tumors were observed in Pelareorep-treated patients (64). Based on these findings, the group tested the efficacy of combined PD-1 inhibition and Pelareorep in mice bearing GL261 glioma and found improved animal survival compared with virotherapy alone (64). In a phase I trial among 6 pediatric patients with refractory malignant brain tumors, intravenous Pelareorep has been assessed in combination with granulocyte-macrophage colony-stimulating factor to enhance systemic viral delivery (NCT02444546) (65). One incidence of dose-limiting toxicity (i.e., hyponatremia) was observed, but maximal tolerable dose was not reached (66). Disease progression was observed in all patients with a median survival of 3.6 months.

### 2.4 Measles virus

Oncolytic measles virus developed from the attenuated Edmonston vaccine strain preferentially infects cells that overexpress CD46, which is typical for many malignancies including glioblastoma (67). In immunocompetent mice bearing orthotopic GL261 glioma, intralesional injection of oncolytic measles virus enhanced infiltration of CD8 + T cells and monocytes and increased expression of PD-L1 in tumors (68). When virotherapy was combined with a PD-1 inhibitor, long-term survival (>120 days) was observed in 60% of the mice, whereas either treatment alone could not sustain animal survival for more than 45 days (68). In contrast, athymic mice that had deficient T cell responses perished within 25 days after tumor challenge even with combination therapy, suggesting a pivotal role of T cells in mediating the therapeutic effect of oncolytic measles virus (68). The safety of oncolytic measles virus has been validated in recurrent glioblastoma patients in a phase I clinical trial, and the median survival after virotherapy was 11.6 months (NCT00390299) (69). The regimen coupling checkpoint blockade and oncolytic measles virus has yet to be translated into the clinic.

### 2.5 Poliovirus

PVSRIPO is a recombinant poliovirus derived from the live-attenuated Sabin vaccine strain (70). The CD155 receptor for poliovirus is expressed on various cancer cells including glioma cells, as well as on myeloid cells such as microglia and macrophages (71). In contrast to other oncolytic viruses, PVSRIPO primarily infects antigen-presenting myeloid cells within the tumor microenvironment, which further recruit and activate tumor antigen-specific T cells through persistent type I/III interferon activation to exert an anti-tumor effect (71, 72). In an immunocompetent murine CT2A glioma model, PVSRIPO treatment caused marked microglia activation throughout the mouse brain, followed by increased infiltration of activated lymphocytes and upregulation of PD-L1 within the tumor microenvironment (73). Although PVSRIPO treatment alone only transiently reduced the tumor size, combination with an anti-PD-L1 antibody resulted in a significant remission rate of 36% (73). Treatment with PVSRIPO in recurrent glioblastoma patients led to 21% of survival at 36 months, remarkably higher than the historical control of 4% (74). Conversely, the median overall survival of PVSRIPO-treated patients was 12.5 months, which was comparable to the historical control of 11.3 months (74), suggesting a potentially patient-specific therapeutic response. Combination of PVSRIPO with pembrolizumab has been assessed in recurrent glioblastoma patients in a phase II trial (NCT04479241) (75), but detailed results have not been published.

### 2.6 Other viruses

Efficacy of several other oncolytic viruses have also been documented in HGG patients. In a small cohort of 14 HGG patients, seven remained alive by the time of report after treatment with an attenuated Newcastle disease virus named MTH-68/H, amounting to 4 to 7 years of survival in four patients (76). However, immune responses to MTH-68/H treatment in patients were not characterized (76). Inoculation with ParvOryx, a rat H-1 parvovirus, extended the median overall survival of 18 recurrent glioblastoma patients to 15.5 months (77). Infiltration of activated CD8 + T cells was found in subsequently resected tumors, although the status of immune checkpoint expression in the tumor microenvironment was unknown (77). One clinical trial assessing a modified vaccinia virus TG6002 in recurrent glioblastoma patients was initiated in 2017 (NCT03294486), but no results are available. Noteworthily, although clinical applications have yet to transpire, Zika virus and live-attenuated Japanese encephalitis virus have been shown to increase intratumoral infiltration of activated T cells, upregulate PD-1 expression on CD4 + (Japanese encephalitis virus) and CD8 + T cells (Zika virus), reduce immunosuppressive regulatory T cells and myeloid-derived suppressor cells, and extend animal survival in murine glioma models that synergizes with checkpoint blockade (78–80), which represent novel development for oncolytic virotherapy.

## 3 Discussion

Various types of oncolytic viruses had significant therapeutic effects in HGG-bearing animal models (22, 46, 61, 67, 77). However, the efficacy of oncolytic virotherapy in HGG patients has been limited in most clinical trials without any substantial improvement in the median overall survival (21, 25, 43, 48, 63). One reason for this might be the suboptimal viral dosages or a lack of multifocal inoculation. Based on preclinical studies, therapeutic effects of oncolytic viruses were found to be dose-dependent (81, 82). Since the primary aim of most clinical trials was to assess the safety profiles of oncolytic viruses in HGG patients, the optimal therapeutic dosages were largely undefined (25, 43, 64, 74). Although adverse reactions such as pyrexia, nausea, headaches, and seizures were common following virotherapy, most events were more likely to be ascribed to the malignancy itself rather than the viral agents (25, 48, 74). The maximum tolerated dose was, thus, not reached in most trials (25, 43, 64, 74). Alternatively, given preclinical evidence suggesting the development of tumor-specific immunological memory after oncolytic virotherapy (35, 55, 83), viral rechallenge or long-term repeated treatment may further promote anti-tumor immune responses (76). Therefore, future trials could expand upon the viral dosage and dosing frequency to enhance the overall efficacy in HGG treatment. Whereas an higher viral dose may trigger immunosuppressive macrophage infiltration (60), counteracting strategies such as incorporating alendronate into the virus construct are under investigation (84). Timing of virotherapy initiation and of co-treatment also warrants exploration. Early initiation of virotherapy prior to radiotherapy may enhance glioma cell susceptibility (85), although clinical data comparing treatment outcomes remain scarce (60).

Another factor that might have undermined the effectiveness of oncolytic virotherapy in HGG patients is the intratumoral and intertumoral molecular heterogeneity, best characterized in glioblastoma (86). Single-cell RNA sequencing has revealed that each primary glioblastoma from patients contains a spectrum of tumor subtypes that differentially express distinct transcriptional programs (87), which could not be recapitulated in preclinical models established from glioblastoma cell lines (e.g., U87). This is important because glioblastoma cells in patients may not uniformly possess the molecular machineries required for preferential viral replication, and thus treatment resistance may arise. Moreover, pediatric HGG may pose unique challenges to oncolytic virotherapy driven by different cancer cell states and immune cell compositions from adult tumors, and heightened immunosuppressive responses to therapy (88, 89), although limited clinical experiences in pediatric patients suggest adequate immune cell infiltration (27) and potential survival benefit (60) following virus treatment. To better capture the heterogeneous nature of HGG, future studies could utilize patient-derived tumor models including xenografts and three-dimensional organoids when evaluating the efficacy of oncolytic viruses (90). On the other hand, there have been some cases where virus-treated HGG patients had sustained remission (21, 26, 74, 76), suggesting a patient-specific therapeutic response. In this regard, defining prognostic factors in HGG patients for oncolytic viruses and careful selection of viral agents might be crucial to improving patients’ prognoses on an individual basis. Stratified prognostic analysis has been limited in clinical trials so far due to primary focus on safety, small cohort size, incomplete molecular profiles, and variable additional treatment. In two studies where stratification was performed, *IDH1* status, especially at recurrence, *MGMT* status, *TP53*, *PTEN*, and *RB1* mutation did not affect therapeutic response to oncolytic viruses (31, 53). Baseline PD-1 expression and intermediary immune cell enrichment within the tumor appeared to be associated with longer patient survival when oncolytic virotherapy was combined with immune checkpoint blockade (53). Studies that further stratify HGG on histologic and molecular basis are needed to identify patient factors that predict therapeutic responses. Ongoing tissue and laboratory monitoring concurrent with virotherapy may also help discover prognostic biomarkers for better treatment adjustment. Whereas decrease in peripheral T cell receptor diversity and a higher clonality after virotherapy has been shown to correlate with progression free survival (51), associations between survival and post-treatment titers of virus neutralizing antibodies or virus clearance rate within tumors have been inconsistent (25, 38, 51, 60).

A third reason might be the inadequacy of virotherapy-induced immunostimulation in HGG patients. In both preclinical and clinical studies, increased infiltration of cytotoxic T cells and M1-polarization of tumor associated macrophages were observed within tumors following virotherapy (27, 48, 49, 64, 68), corroborating the presence of immunostimulation. Radiographic evidence suggestive of immune activation and therapeutic responses was noted in some patients where the size of the enhancing lesion increased shortly after the initiation of virotherapy for up to months until the onset of a steady tumor shrinkage, a process called pseudoprogression (48, 74). However, a lack of durable responses in the majority of patients signifies that the immunostimulatory effects of oncolytic viruses as a monotherapy may still be insufficient (48, 64, 68, 74), and further immune modulation may be necessary. In light of this idea, newer generations of oncolytic viruses have been armed with pro-inflammatory cytokines (e.g., IL-2 and IL-12 in G47Δ-mIL2 and G47Δ-mIL12, respectively, interleukin 27 in HSV-IL27, class II major histocompatibility complex trans-activator in Ad-CIITA) or co-stimulatory ligands (e.g., OX40L in DNX-2440), or co-administered with immune stimulatory ligands (e.g., CD40 agonists), which has resulted in enhanced efficacy in preclinical HGG models compared with parental viruses (34, 55, 91–93). In addition, virotherapy-induced expression of inhibitory immune checkpoints such as PD-1 on tumor infiltrating lymphocytes and PD-L1 on tumor cells supports the notion of synergizing oncolytic viruses with checkpoint inhibitors (47, 64, 68, 94, 95). Preclinical studies have found this combination to be more efficacious than virotherapy alone in murine HGG models (35, 47, 55, 64, 68, 96, 97). In clinical settings, supplementing DNX-2401 with a PD-1 inhibitor appeared to have improved the survival of recurrent HGG patients (52, 53), compared with DNX-2401 alone in a separate trial (48). Given that the combination therapy has only been evaluated in one clinical trial (53), future studies could appraise the synergistic effects of oncolytic viruses and checkpoint inhibitors using different agents in HGG patients. Nevertheless, differential immune landscapes of HGG at the baseline and treatment-induced recruitment of immunosuppressive myeloid cells may also influence responses to virotherapy and immune checkpoint blockade (53). Additional therapeutics to maximize immune responses, such as with epigenetic agents and NOTCH signaling pathway inhibitors, may be necessary to enhance treatment efficacies (98, 99).

**Figure 2:**
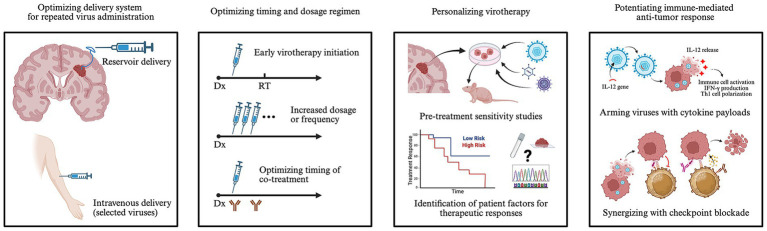
Schematic of potential strategies to improve efficacy of virotherapy in HGG treatment. Use of an Ommaya reservoir or intravenous route can simplify virus delivery, especially for repeated administration. Early initiation of virotherapy after HGG diagnosis (Dx), e.g., prior to radiotherapy (RT), and increased dosage or frequency may improve treatment responses. Optimal timing for co-treatment warrants exploration. Pre-treatment *in vitro* and *in vivo* sensitivity assays using patient-derived HGG cultures may help personalize virus selection. Characterizing baseline genetic and histologic features and ongoing biomarker changes in cerebrospinal fluid during treatment may unveil prognostic factors and facilitate treatment adjustment. Arming oncolytic viruses with cytokine payloads such as IL-12 and synergizing with checkpoint blockade may further activate anti-tumor immune responses. Created in BioRender. Qian, Z. (2026), https://BioRender.com/j8xvi0c.

A significant translational gap exists between the preclinical efficacy and clinical application of combined oncolytic viruses and immune checkpoint inhibitors, and more trial designs are urgently needed. Establishing biobanks for patient-derived glioma samples could offer further preclinical insights to support clinical translation. Additionally, the relatively low incidence of HGG underscores the importance of multi-center collaboration. The immunostimulatory and anti-tumor effects of virus treatment may diminish over time (73), and thus repeated virus dosing may be necessary in future trials. Convection-enhanced delivery improves efficacy compared to simple infusions in preclinical models (100), but is not permissive to recurring treatment (50, 101). In contrast, Ommaya/Rickham reservoirs, with intratumorally-directed catheter tips (60), and an intravenous route (66, 76, 77) have been shown clinically feasible and safe delivering selected viruses, which may significantly facilitate repeated dosing (Figure 2). Efficiency and efficacy across delivery modes warrant further clinical investigation. Moreover, the optimal sequence for virotherapy and immune checkpoint blockade is controversial and may be influenced by virus type, delivery method, dosage, frequency, checkpoint type, tumor type, presence of cytokine payload, and additional therapies (102, 103). In preclinical glioma models, both concurrent (35, 73) and sequential regimen (i.e., virus priming followed by checkpoint inhibition) (47, 55, 64) have shown efficacy. Thus far, clinical studies have primarily employed a sequential regimen spaced by 1–4 weeks (NCT05084430, NCT04479241) (53). Further exploration of intratumoral temporal changes in the immune infiltration and checkpoint expression following virotherapy and their associations with therapeutic efficacy will be crucial to defining the optimal treatment sequence. Importantly, prognostic factors in HGG patients that predict therapeutic responses to virotherapy remain largely unknown. Whereas conventional molecular tumor markers have failed to demonstrate prognostic values in a few studies (31, 53), evaluation of immunologic parameters associated with antigen-specific immune activation such as T cell receptor diversity and clonality may better predict therapeutic responses (51). Intricate study designs with pre-surgical virotherapy by up to 2 weeks have been shown feasible (25, 48, 69, 77) and may facilitate systemic prognostic analysis based on matched pre- and post-treatment tumor samples to guide patient selection for future trials. Cellular and molecular characterization at different stages (e.g., baseline biopsy during pre-surgical virus injection, immediate post-treatment period during surgical resection, and tumor progression when re-resection is appropriate) may also help dissect longitudinal tumor and immune landscapes for development of further combination treatment.

## 4 Conclusion

The therapeutic benefits of oncolytic viruses alone in HGG patients have been limited in most clinical trials, and further improvement in treatment regimen is urgently needed. However, both preclinical and clinical evidence exist that suggest activation of tumor-specific immune responses following oncolytic virotherapy. Moreover, in limited preclinical studies and one clinical trial, combination of oncolytic virotherapy and checkpoint blockade had synergistic effects and improved subject survival, while maintaining an amenable safety profile. Hence, therapy-induced accrual of tumor infiltrating lymphocytes and elevated expression of inhibitory immune checkpoints within tumors provide the basis for the use of checkpoint inhibitors in concert with oncolytic viruses. Given the highly heterogeneous nature of HGG, the treatment regimen should be multimodal to maximize efficacy. Future studies could explore the synergistic effects of more oncolytic viruses and checkpoint inhibitors in HGG patients. Optimizing timing of virotherapy and co-treatment, improving virus dosage and frequency, and personalizing virus selection are also important directions for further research that require multi-center collaboration. More importantly, a better understanding of the tumor immune landscape following oncolytic virotherapy is needed to help establish better combination regimens to improve the clinical outcomes of HGG patients.
